# Screening Adults for HIV Testing in the Outpatient Department: An Assessment of Tool Performance in Malawi

**DOI:** 10.1007/s10461-021-03404-8

**Published:** 2021-08-11

**Authors:** Corrina Moucheraud, Risa M. Hoffman, Kelvin Balakasi, Vincent Wong, Maria Sanena, Sundeep Gupta, Kathryn Dovel

**Affiliations:** 1 Department of Health Policy and Management, Fielding School of Public Health, University of California, 650 Charles E. Young Dr. S., 31-235A, Los Angeles, CA 90095 USA; 2 Division of Infectious Diseases, David Geffen School of Medicine, University of California, Los Angeles, CA USA; 3 Partners in Hope, Lilongwe, Malawi; 4 USAID Global Health Bureau, Washington, DC USA

**Keywords:** Screening, HIV testing, Health systems, Malawi

## Abstract

Little is known about screening tools for adults in high HIV burden contexts. We use exit survey data collected at outpatient departments in Malawi (n = 1038) to estimate the sensitivity, specificity, negative and positive predictive values of screening tools that include questions about sexual behavior and use of health services. We compare a full tool (seven relevant questions) to a reduced tool (five questions, excluding sexual behavior measures) and to standard of care (two questions, never tested for HIV or tested > 12 months ago, or seeking care for suspected STI). Suspect STI and ≥ 3 sexual partners were associated with HIV positivity, but had weak sensitivity and specificity. The full tool (using the optimal cutoff score of ≥ 3) would achieve 55.6% sensitivity and 84.9% specificity for HIV positivity; the reduced tool (optimal cutoff score ≥ 2) would achieve 59.3% sensitivity and 68.5% specificity; and standard of care 77.8% sensitivity and 47.8% specificity. Screening tools for HIV testing in outpatient departments do not offer clear advantages over standard of care.

**Supplementary Information:**

The online version contains supplementary material available at 10.1007/s10461-021-03404-8.

## Introduction

In order to achieve control of the HIV epidemic, UNAIDS has set a series of ambitious targets striving for 95% of individuals living with HIV to know their status, 95% of those to initiate ART, and 95% of those on ART to reach viral suppression [1]. There are, however, persistent gaps in identifying people living with HIV. Approximately 11% of people living with HIV in Eastern and Southern Africa do not know their HIV status [2]. Although there has been progress in the past decade, it has been slow and variable by region [3]. Additionally, as HIV testing coverage increases and HIV incidence declines [4], more resources are needed to diagnose each new case [5, 6].

The global community is thus seeking innovative ways to more efficiently identify people who should be tested for HIV—including new technologies (self-testing) [7], new operational approaches to testing (e.g., index partner testing, community-based contact tracing, and social or risk network testing) [8–10], and the use of screening tools to identify those most in need of testing [11]. Clinical HIV guidelines in many African settings currently recommend testing all pregnant women, individuals with newly-diagnosed tuberculosis, and all sexually active adults annually and whenever they seek care for a sexually transmitted infection (STI) [4]—but, risk assessment through screening may be more sensitive and/or specific than this standard of care (SOC). Screening tools have previously been developed for pediatric HIV diagnosis in sub-Saharan Africa [12, 13], and may be a useful tool to prioritize high-risk adults for testing. If a screening tool has sufficiently high sensitivity and specificity for HIV infection, it can guide how to use limited testing resources including supplies, staff and space.

Outpatient departments (OPDs) offer an opportunity for case finding [14] as many adults present regularly to OPDs for acute services [15, 16]. Facility-based testing also offers advantages through better linkage to ART services and cost savings compared to community-based testing [8, 17, 18]; however traditional provider-initiated-testing-and-counseling (PITC) has low coverage in OPD settings due to overcrowded and understaffed departments [19, 20] and has traditionally demonstrated low HIV positivity rates among those tested [14]. The “yield” (positivity among those tested) of current diagnostic approaches in Africa is also declining more broadly [21]. An HIV screening tool specific to OPD settings may improve testing efficiencies. However, understanding sensitivity and specificity of the tool is critical, as the introduction of a new workflow in OPD will impose additional time required for patients and staff. Therefore, assessing tool performance relative to SOC is an important first step in the decision to implement an HIV screening tool in OPD settings.

The nature of screening tool questions for crowded OPD settings also requires careful consideration. For example, questions regarding sexual risk behavior may unintentionally stigmatize certain individuals and populations. Although questions about recent sexual behavior may be predictive of HIV status [22, 23], these may not be feasible to implement in busy OPD settings due to lack of private spaces to ask sensitive questions and obtain accurate responses [19, 22]. For example, during screening for STI or HIV testing in the United States, health care providers in busy emergency departments were routinely less likely to ask questions regarding sexual health as compared to other non-sensitive topics [22]. In order to develop more efficient strategies for HIV testing in OPD settings, more evidence is needed about how screening tools perform with and without sensitive questions, and compared to SOC.

The goal of our study was to assess whether a screening tool could increase the efficiency of HIV testing among adults seeking care in OPD settings in Malawi, as compared to SOC. We compare the estimated performance of two versions of a screening tool—one with sexual behavior questions (i.e., full tool) and one without (i.e., reduced tool)—to assess the sensitivity and specificity of potential screening tools in OPD settings.

## Methods

### Parent Study

Data were collected during exit surveys from a large cluster-randomized trial which aimed to assess the impact of facility HIV self-testing among adult outpatients compared to PITC. Health facilities were randomized to one of three arms (five facilities per arm) using constrained randomization based on region and facility type. The intervention is described in more detail elsewhere [24, 25]. For this secondary analysis, we used data from the HIV self-testing arm (five facilities) due to high HIV testing coverage within the arm, therefore with sufficient numbers of outpatients tested in order to reach sufficient power for this sub-analysis. In the HIV self-testing arm, outpatients were approached in outpatient waiting spaces and encouraged to test for HIV if they were > 15 years of age, had never received an HIV-positive diagnosis, and had not tested HIV-negative within the last 3 months (or never tested). Facilities were equally spread across central and southern Malawi and varied in facility type: one district hospital; one mission hospital; and three large health centers.

### Data Collection

A subset of outpatients at participating facilities were recruited using a systematic sampling strategy. Research staff recruited every tenth outpatient exiting the outpatient department to complete an exit survey. Eligibility criteria were: ≥ 15 years of age; accessed and completed all outpatient services to be received that day. Oral consent was attained and exit surveys completed in private, quiet locations at the health facility. The survey was administered by trained research staff, who asked survey questions aloud and recorded the respondents’ answers on a tablet using data collection software. From the five HIV self-testing arm facilities, 2183 adult outpatients were approached to complete exit surveys about their testing experience and 2097 respondents were eligible and completed a survey (September 2017–February 2018). This analysis includes survey data from individuals who reported testing for HIV on the day of enrollment, and reported never testing HIV-positive prior to the day of enrollment (in order to examine screening tool performance in the context of new HIV diagnoses).

### Survey Tool

The exit survey tool was created based on conceptually-driven hypotheses about associations with HIV status, including: (1) sociodemographic variables; (2) previous use of HIV services and test results; (3) risky sexual behavior in the past 12 months; (4) health services received that day; and (5) use of HIV testing and result of any HIV test received that day. All surveys were completed in the local language (Chichewa) and lasted approximately 20 min on average.

### Data Analysis

We used questions from the exit survey to approximate a screening tool; questions that captured factors hypothetically associated with HIV risk were included in the analysis.

#### Operationalizing the Screening Tool

We included seven variables in the full screening tool. We included suspected sexually transmitted infections (STI) (defined as attending the facility for an STI) because STIs are a known risk factor for seroconversion [26], and attending the facility for malaria-like symptoms since malaise and fever have previously been associated with recent HIV seroconversion in similar settings [27]. We also included “overlooked” groups who do not have a standard entry point for HIV testing: women below the age of 25 (since women often test during pregnancy at antenatal visits), and men over the age of 24 because men almost exclusively seek care via OPD for acute needs [15, 28], therefore if they are not engaged at OPD they may not be reached. These groups are also important for the evolving HIV epidemic in the region: both men and young women have higher HIV positivity, test less often and may not be well-served by current HIV testing modalities [3, 29–31]. Finally, never tested for HIV or tested > 12-months ago was included as SOC. The response to each tool question was scored as a 0/1 (no/yes), and these responses were summed to create the total screening tool score.

We also dichotomized two questions with continuous response options (number of recent health visits, and number of recent sexual partners). Cut points were selected based on association with HIV status: we assessed the “inflection point” at which these items became strongly associated with HIV positivity (e.g., having two or more sexual partners was not significantly associated but having three or more sexual partners was).

#### Analyzing Screening Tool Performance

The analysis aimed to estimate the performance of screening tools, comprised of survey questions as described above, to predict HIV positivity among outpatients testing for HIV during routine outpatient services. We analyzed the data in two ways (1) using all relevant questions from the survey (items 1–7 in Table 1) and (2) using a reduced tool that excluded questions about number of sexual partners and sex outside of marriage (items 1–5 in Table 1). The SOC approach to HIV testing was captured by items 1 and 2 in Table 1.

**Table 1:** Screening tool questions

Question(s)		Tool*
HIV testing history	1. Never been tested for HIV before Or, Tested previously for HIV but 12 + months ago	F,R,S
Medical history	2. At health facility today for suspected STI	F,R,S
	3. At health facility today for suspected malaria	F,R
	4. Received health services 4 + times during the last 6 months (excluding today's visit)	F,R
Demographic risk	5. “Overlooked” groups: female and < 25 Or, male and > 24	F,R
Behavioral risk	6. Have had 3 + sexual partners in the past 12 months	F
	7. Had sex without a condom with someone besides spouse in the past 12 months	F

*Full (F), Reduced (R), Standard of care (S)

Of the 2097 outpatients who completed a survey, 1034 were excluded from this analysis due to not testing for HIV on the day of enrollment, 11 were excluded because they had previously tested HIV-positive and 14 were excluded because they either refused to disclose the test result or reported not knowing the test result. All included respondents (n = 1038) had complete data for the survey questions used to calculate the screening tool score. This sample size is sufficient for assessing a tool with 40% sensitivity (with specificity up to 99%) given estimated adult HIV prevalence of 9% [32].

We used logistic regression to assess the relationship between HIV status and respondent characteristics (age and sex), response to each screening tool item, and total screening tool scores (full and reduced tool). We calculated the sensitivity, specificity, negative and positive predictive values (NPV and PPV, respectively) for each screening tool item and total screening tool scores (full and reduced) using Stata v14. The optimal screening tool cutoff was identified by plotting the receiver operating characteristic curve and estimating the partial area under the curve (correcting for ties).

### Ethical Review

The parent trial including the exit survey methodology and instrument received ethical approval from the Malawi National Health Sciences Review Committee and the University of California Institutional Review Board.

## Results

As described above, a total of 1038 adults participated in an exit survey, used a HIV self-test kit, and had a known HIV test result so are included in this analysis. Most participants were female (n = 684, 65.9%) and the mean age was 32.2 years (SD 13.0) (Table 2). Overall, 2.6% of respondents (n = 27) tested HIV-positive. Approximately half of respondents had been tested > 12 months ago (or never tested) (n = 540, 52.0%), 44.2% were a member of an “overlooked” group (women < 25 and men > 24 years) (n = 459); and just under one-quarter reporting having ≥ 3 sexual partners in the past year (n = 247, 23.8%). The average score was 1.5 (SD 1.1) for the full screening tool (out of a maximum possible score of 7) and 1.2 (SD 0.8) for the reduced screening tool (out of a maximum possible score of 5).

**Table 2:** Characteristics of study participants

	Overall (n = 1038)	Among those HIV-negative (n = 1011)	Among those HIV-positive (n = 27)	Odds ratio^‡^ (95% CI)	p-value
Age, mean (SD)	32.2 (13.0)	32.1 (13.1)	34.1 (11.0)	1.01 (0.99, 1.03)	0.33
Gender, n (%)					
Female	684 (65.9%)	667 (66.0%)	17 (63.0%)	1.14 (0.52, 2.52)	0.75
Male	354 (34.1%)	344 (34.0%)	10 (37.0%)		
Screening items, n (%)					
“Overlooked” groups (women 15–24, men 25 +)	459 (44.2%)	443 (43.8%)	16 (59.3%)	1.86 (0.86, 4.06)	0.12
Tested ≥ 12 months ago or never tested	540 (52.0%)	520 (51.4%)	20 (74.1%)	2.70 (1.13, 6.44)	0.03
Came in for a suspected STI	18 (1.7%)	16 (1.6%)	2 (7.4%)	4.98 (1.08, 22.82)	0.04
≥ 4 recent health consultations (last 6 months)	127 (12.2%)	120 (11.9%)	7 (25.9%)	2.60 (1.08, 6.28)	0.03
Came in for suspected malaria (i.e., fever, malaise, etc.)	76 (7.3%)	72 (7.1%)	4 (14.8%)	2.27 (0.76, 6.74)	0.14
≥ 3 recent sexual partners (last 12 months)	92 (8.9%)	86 (8.5%)	6 (22.2%)	3.07 (1.21, 7.82)	0.02
Recent condomless sex with a non-stable partner (last 12 months)	247 (23.8%)	235 (23.2%)	12 (44.4%)	2.64 (1.22, 5.72)	0.01
Full screen^ score, mean (SD)	1.5 (1.1)	1.5 (1.0)	2.5 (1.4)	2.11 (1.50, 2.98)	< 0.001
Reduced screen^^ score, mean (SD)	1.2 (0.8)	1.2 (0.8)	1.8 (1.0)	2.43 (1.50, 3.96)	< 0.001
Standard of care^^^ score, mean (SD)	0.5 (0.5)	0.5 (0.5)	0.8 (0.4)	3.20 (1.28, 8.00)	0.01

^‡^Odds ratios include robust standard errors

^Full tool includes: “overlooked groups,” tested ≥ 12 months ago or never; came in for STI; ≥ 4 recent health consultations; came in for malaria; ≥ 3 recent sexual partners; recent condomless sex with a non-stable partner. Possible score ranges 0–7

^^Reduced tool includes: “overlooked groups,” tested ≥ 12 months ago or never; came in for STI; ≥ 4 recent health consultations; came in for malaria. Possible score ranges 0–5

^^^ Standard of care is tested ≥ 12 months ago or never; and/or came in for STI. Possible score ranges 0–1

HIV positivity was significantly and strongly associated with screening questions (Table 2). Among those with a suspected STI, 11.1% tested HIV-positive (versus only 2.5% of those who attended OPD for other reasons); and 6.5% of people reporting > 3 sexual partners in the past 12-months were HIV-positive (versus 2.2% of people with fewer sexual partners). In adjusted models that included covariates for age and sex, the odds of being HIV-positive was approximately 2–4 times higher for all screening questions compared to respondents who answered “no” to the same question (Appendix Table A1). HIV positivity increased with higher screening tool score (both full and reduced tool) (Appendix Table A2).

Table 3 examines the specificity and sensitivity of each item and of the full and reduced (without sexual behavior questions) screening tools. The most sensitive items were: tested ≥ 12 months ago or never tested (sensitivity 74.1%, 95% CI 53.7–88.9%), and being in an “overlooked” group (sensitivity 59.3%, 95% CI 38.8–77.6%). However, both had low specificity (56.2% and 48.6%, respectively). The items with highest specificity were: suspected STI (specificity 98.4%, 95% CI 97.4–99.1%); suspected malaria (specificity 92.9%, 95% CI 91.1–94.4%); and > 3 recent sexual partners (specificity 91.5%, 95% CI 89.6–93.1%)—however, these items had low sensitivity (7.4%, 14.8%, 22.2%, respectively).

**Table 3:** Screening item and tool performance

	Sensitivity (95% CI)	Specificity (95% CI)	PPV (95% CI)	NPV (95%) CI)
“Overlooked” groups (women 15–24, men 25 +)	59.3% (38.8, 77.6%)	56.2% (53.1, 59.3%)	3.5% (2.0, 5.6%)	98.1% (96.6, 99.0%)
Tested ≥ 12 months ago or never	74.1% (53.7, 88.9%)	48.6% (45.4, 51.7%)	3.7% (2.3, 5.7%)	98.6% (97.1, 99.4%)
Came in for a suspected STI	7.4% (0.9, 24.3%)	98.4% (97.4, 99.1%)	11.1% (1.4, 34.7%)	97.5% (96.4, 98.4%)
≥ 4 recent health consultations	25.9% (11.1, 46.3%)	88.1% (86, 90.1%)	5.5% (2.2, 11%)	97.8% (96.6, 98.7%)
Came in for suspected malaria	14.8% (4.2, 33.7%)	92.9% (91.1, 94.4%)	5.3% (1.5, 12.9%)	97.6% (96.4, 98.5%)
≥ 3 recent sexual partners	22.2% (8.6, 42.3%)	91.5% (89.6, 93.1%)	6.5% (2.4, 13.7%)	97.8% (96.6, 98.6%)
Recent condomless sex with a non-stable partner	44.4% (25.5, 64.7%)	76.8% (74, 79.3%)	4.9% (2.5, 8.3%)	98.1% (96.9, 98.9%)
Full screening tool score^				
Score ≥ 1	92.6% (75.7, 99.1%)	16% (13.8, 18.4%)	2.9% (1.9, 4.2%)	98.8% (95.7, 99.9%)
Score ≥ 2	70.4% (49.8, 86.2%)	55.8% (52.7, 58.9%)	4.1% (2.5, 6.3%)	98.6% (97.3, 99.4%)
Score ≥ 3	55.6% (35.3, 74.5%)	84.9% (82.5, 87%)	8.9% (5.1, 14.3%)	98.6% (97.6, 99.3%)
Score ≥ 4	22.2% (8.6, 42.3%)	96.4% (95.1, 97.5%)	14.3% (5.4, 28.5%)	97.9% (96.8, 98.7%)
Score ≥ 5	7.4% (0.9, 24.3%)	99.4% (98.7, 99.8%)	25% (3.2, 65.1%)	97.6% (96.4, 98.4%)
Reduced screening tool score^^				
Score ≥ 1	92.6% (75.7, 99.1%)	21.2% (18.7, 23.8%)	3.0% (2.0, 4.5%)	99.1% (96.7, 99.9%)
Score ≥ 2	59.3% (38.8, 77.6%)	68.5% (65.6, 71.4%)	4.8% (2.8, 7.7%)	98.4% (97.2, 99.2%)
Score ≥ 3	25.9% (11.1, 46.3%)	94.6% (93, 95.9%)	11.3% (4.7, 21.9%)	98% (96.9, 98.7%)
Score ≥ 4	3.7% (0.1, 19%)	99.9% (99.5, 100%)	50% (1.3, 98.7%)	97.5% (96.3, 98.4%)
Standard of care^^^ score				
Score ≥ 1	77.8% (57.7, 91.4%)	47.8% (44.7, 50.9%)	3.8% (2.4, 5.8%)	98.8% (97.3, 99.5%)

^Full tool includes: “overlooked groups,” tested ≥ 12 months ago or never; came in for STI; ≥ 4 recent health consultations; came in for malaria; ≥ 3 recent sexual partners; recent condomless sex with a non-stable partner. Possible score ranges 0–7

^^Reduced tool includes: “overlooked groups,” tested ≥ 12 months ago or never; came in for STI; ≥ 4 recent health consultations; came in for malaria. Possible score ranges 0–5

^^^Standard of care is tested ≥ 12 months ago or never; and/or came in for STI. Possible score ranges 0–1

**Fig. 1:**
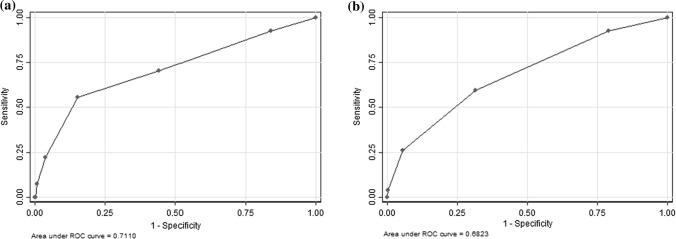
Receiver operating characteristic curve for identifying HIV-positive adults using the full screening tool (**a**, left) and using the reduced screening tool (**b**, right)

For the full screening tool, examining the score as a continuous measure shows that at a cutoff of ≥ 1, the tool specificity was 16.0% (95% CI 13.8–18.4%) and sensitivity was 92.6% (95% CI 75.7–99.1%) for HIV test positivity. Using a cutoff score of ≥ 2 the specificity was 55.8% (95% CI 52.7–58.9%) and sensitivity was 70.4% (95% CI 49.8–86.2%). At a cutoff score of ≥ 3 the specificity was 84.9% (95% CI 82.5–87.0%) and sensitivity was 55.6% (95% CI 35.3–74.5%)—and this is the optimal cutoff score based on the ROC (Fig. 1a) (partial area under ROC curve 0.70, 95% CI 0.61–0.80). The positive predictive value at the ≥ 3 item cutoff score is 8.9% and the negative predictive value is 98.6%.

The reduced screening tool had improved specificity when compared to the full tool at all cutoff points, but sensitivity was slightly lower. At a score of ≥ 1, the specificity was 21.2% (95% CI 18.7–23.8%) and sensitivity was 92.6% (95% CI 75.7–99.1%) for HIV test positivity. At a score of ≥ 2 the specificity was 68.5% (95% CI 65.6–71.4%) and sensitivity 59.3% (95% CI 38.8–77.6%), with a positive predictive value of 4.8% and a negative predictive value of 98.4%; and this is the optimal cutoff with a partial area under ROC curve of 0.64 (95% CI 0.54–0.74) (Fig. 1b).

The SOC screen—i.e., tested > 12 months ago or never, and/or presented with an STI—had a sensitivity of 77.8% (95% CI 57.7–91.4%) and a specificity of 47.8% (95% CI 44.7–50.9%). SOC screening had a positive predictive value of 3.8% (95% CI 2.4–5.8%) and a negative predictive value of 98.8% (95% CI 97.3–99.5%).

## Discussion

We found that in OPD settings neither a tool with sexual behavior questions nor one excluding sexual behavior questions performed better than the standard of care approach to HIV testing in Malawi, and we could not identify a set of questions that was both highly sensitive and specific. The full screening tool at an optimal score of ≥ 3 would achieve 55.6% sensitivity and 84.9% specificity for HIV positivity among adult outpatients in Malawi. The reduced tool at an optimal score of ≥ 2 would achieve 59.3% sensitivity and 68.5% specificity, while standard of care of having at least one of two questions would achieve a sensitivity of 77.8% and specificity of 47.8%. Although standard of care screening had the highest sensitivity, it also had lower specificity than either full or reduced tools at their optimal cutoff scores. Similar to other literature, newly-diagnosed HIV infection was highly correlated with having suspected STI and ≥ 3 sexual partners in the past 12-months, [33–35], although adding these questions did not improve screening tool performance.

Our findings highlight the challenging trade-offs in finding an approach that is efficient (i.e., tool sensitivity) and still reaches the majority of unidentified individuals living with HIV (i.e., tool specificity). Given the adult HIV prevalence in Malawi (8.9%) [32], the treatment-adjusted prevalence of 1.7% [36], and the fact that less than 23% of individuals living with HIV were undiagnosed at the time of our study [37], for each 1000 adult outpatients screened, the full screening tool at an optimal score (≥ 3) would result in 162 people being tested for HIV, and 14 of these people would test positive; while among those not tested, there would be 12 HIV-positive people left undiagnosed. If 1000 people were screened using the reduced tool, using its optimal score (≥ 2), 322 would undergo an HIV test, among which 15 people would be diagnosed as HIV-positive and 11 HIV-positive people would be left undiagnosed.

One aim of screening tools is to save resources by prioritizing who gets tested. To achieve this, tools must have high sensitivity and specificity because their implementation adds burden for facility staff who are already under significant constraints due to high patient volume and limited resources in OPD settings. Program data from other screening experiences among outpatients in Malawi indicate that multiple-question screening tools (with sexual behavior questions) require five to seven minutes to administer per person screened [38]. Screening tools that include questions about sexual behavior also require private space for implementation, as well as patient-provider trust in order to promote open, honest responses from outpatients [22, 23].

Screening tool performance is affected by underlying prevalence, and there is an increased likelihood of more false positives in low-prevalence settings, which would affect the screening tool’s predictive values. Therefore, in contexts with controlled or near-controlled epidemics, it may be particularly challenging to identify adult HIV testing screening tools with sufficiently high specificity to offer efficiencies over standard of care, and sufficiently high sensitivity to capture most infected individuals. A recent paper analyzed the performance of a similar adult outpatient screening tool in Kenya, and found that the optimal set of questions (which included both demographic characteristics and sexual risk behavior questions) would reduce the number of people requiring testing by 75%, but would miss approximately half of HIV-positive individuals [33]. Taken together with our findings from Malawi, it is evident that screening tools are not an optimal solution for HIV testing in outpatient departments. As local epidemics evolve, multi-pronged approaches will be increasingly necessary to find the relatively small number of individuals unidentified with HIV.

While screening tools for adults in OPD settings have suboptimal performance, screening tools for pediatric HIV testing in sub-Saharan African OPDs show more promise, with sensitivity of approximately 71–92% and specificity of approximately 32–88% [39]. Pediatric tools may perform better than adult tools in OPD settings because questions related to vertical transmission risk are easier to include in a screening tool, compared to HIV horizontal acquisition risk factors for adults which may encompass a range of sensitive behaviors and exposures. Additionally, the physical manifestations of HIV infection may be easier to detect in children than in adults, for example frequent infections and failure to thrive [40]. Even still, the highest-performing pediatric HIV screening tool identified in a recent systematic review (with sensitivity and specificity ≥ 95%) was quite complex and may be difficult to take to scale. The tool incorporated 17 questions that included locally-relevant risk factors for HIV status such as father’s occupation, location and health status [39]—suggesting that deploying optimal screening tools, even for pediatrics, requires substantial resources including dedicated and trained personnel to administer lengthy questionnaires.

Without a well-performing screening tool, what strategies can increase HIV testing efficiency in OPD settings? Facility HIV self-testing can test many adults without increased personnel or the need for additional infrastructure [25]. HIV self-testing is effective and can provide cost savings for identifying newly-diagnosed adults in OPD as compared to standard blood-based testing [41] – but does require procurement and distribution of a high volume of test kits, which may be challenging in low-resource health systems. Other options might be facility-based testing “campaigns” or targeted time periods of concentrated testing aimed at achieving high coverage with consolidated resources. Reaching the remaining unidentified individuals living with HIV will likely require an approach that combines sustained OPD testing with higher-yield testing strategies, such as index partner testing, leveraging social networks of high-risk populations, and providing work-based testing for men in higher-risk occupations [8–10]. Programs and policymakers should also consider whether ensuring comprehensive HIV testing using SOC in OPDs, despite increasing inefficiencies and cost due to evolution of the HIV epidemic, is simply a necessary cost of sustaining control of a generalized epidemic.

This study has some limitations that should be noted. We performed a post-hoc analysis of a “constructed” hypothetical screening tool based on questions from an exit survey collected as part of a large trial about HIV self-testing. Some key risk factors that may be predictive of HIV positivity among adults—such as region of residence, number of lifetime partners, household wealth, migratory labor, and suspect tuberculosis [42–46]—were not included in the parent study survey tool so could not be analyzed here. There may have been selection bias into participation into the parent study (those who opted to use the HIV self-test), and there may have been response bias to the exit survey questions, especially those about sexual behavior. For example, people may have answered questions about their risk behaviors based on their HIV status, as diagnosed just prior to data collection. Additionally, the survey was asked at the conclusion of each person’s clinical encounter, so their experience at the facility that day—including learning of their HIV status for those newly diagnosed—may have affected their responses to survey questions. Lastly, the scoring algorithm used here is simplistic (summing dichotomous values) and does not consider the relative importance of different factors or how this might affect overall scores, for example through a weighted average. Given the small number of HIV cases in this population and our interest in identifying a screening tool that could be easily implemented in a busy OPD setting, we pursued this less complex approach when designing the screening tool. However, a more nuanced clinical predictive tool, that scores based on strength of association between the factor and the outcome, is worthy of further study particularly in lager populations with higher underlying HIV prevalence.

## Conclusions

While we found screening questions that were highly correlated with HIV status, the screening tools (full or reduced) did not offer compelling advantages to efficiency or feasibility beyond the SOC. These findings, in addition to the added burden and complexities of introducing an adult screening tool within limited-resource OPD settings, and the large heterogeneity in health facility and epidemiological contexts within countries, call into question the use of screening tools for improving the efficiency of HIV testing in OPDs. While identification of an effective screening tool would represent a needed step forward in fighting the HIV epidemic, this study highlights the need for rigorous evidence before introducing such an approach at scale.

## Supplementary Information

Below is the link to the electronic supplementary material.

Supplementary file1 (DOCX 20 kb)
