# Supplementary material for: Screening Adults for HIV Testing in the Outpatient Department: An Assessment of Tool Performance in Malawi
Source: AIDS Behav. 2021 Aug 11;26(2):478–86. doi: 10.1007/s10461-021-03404-8 (PMC8813838; doi:10.1007/s10461-021-03404-8)
Supplement: Supplementary file 1 — Supplementary file1 (DOCX 20 kb) [file 10461_2021_3404_MOESM1_ESM.docx]

Supplemental tables

| **Table A1. Characteristics of study participants: Odds ratios and adjusted odds ratios** | | | | |
| --- | --- | --- | --- | --- |
|  | Odds ratio‡  (95% CI) | p-value | Adjusted odds ratio‡ (95% CI) | p-value |
| Screening items |  |  |  |  |
| “Overlooked” groups (women 15-24, men 25+) | 1.86  (0.86, 4.06) | 0.12 | 2.57  (1.10, 6.00) | 0.03 |
| Tested ≥12months ago or never tested | 2.70  (1.13, 6.44) | 0.03 | 2.60  (1.07, 6.29) | 0.03 |
| Came in for a suspected STI | 4.98  (1.08, 22.82) | 0.04 | 3.90  (0.79, 19.16) | 0.09 |
| ≥4 recent health consultations | 2.60  (1.08, 6.28) | 0.03 | 2.75  (1.15, 6.53) | 0.02 |
| Came in for suspected malaria (i.e., fever, malaise, etc.) | 2.27  (0.76, 6.74) | 0.14 | 2.00  (0.62, 6.49) | 0.25 |
| ≥3 recent sexual partners within 12-months | 3.07  (1.21, 7.82) | 0.02 | 3.22  (1.15, 8.98) | 0.03 |
| Condomless sex with a non-stable partner within 12-months | 2.64  (1.22, 5.72) | 0.01 | 3.93  (1.58, 9.79) | 0.003 |
| Full screen^ score | 2.11  (1.50, 2.98) | < 0.001 | 2.58  (1.74, 3.82) | < 0.001 |
| Reduced screen^^ score | 2.43  (1.50, 3.96) | < 0.001 | 2.69  (1.65, 4.37) | < 0.001 |
| Standard of care^^^ score | 3.20  (1.28, 8.00) | < 0.001 | 3.05  (1.21, 7.71) | 0.02 |

*‡ Odds ratios include robust standard errors. Adjusted odds ratios include covariates for: age (continuous), sex (male/female), educational attainment (none/any primary/any secondary/beyond), married (yes/no), worked in last 7 days (yes/no), number of living children*

*^ Full tool includes: “overlooked groups,” tested ≥12 months ago or never; came in for STI; ≥4 recent health consultations; came in for malaria; ≥3 recent sexual partners; recent condomless sex with a non-stable partner. Possible score ranges 0-7.*

*^^ Reduced tool includes: “overlooked groups,” tested ≥12 months ago or never; came in for STI; ≥4 recent health consultations; came in for malaria. Possible score ranges 0-5.*

*^^^ Standard of care is tested ≥12 months ago or never; and/or came in for STI. Possible score ranges 0-1.*

| **Table A2. Association between screening tools and HIV positivity (n=1038)** | | | | | | | | | | | |
| --- | --- | --- | --- | --- | --- | --- | --- | --- | --- | --- | --- |
|  | **Full screening tool^:** | | | | | **Reduced screening tool^^:** | | | | | |
|  | n (%) HIV positive | OR‡ (95% CI) | p-value | Adjusted OR‡  (95% CI) | p-value |  | n (%) HIV positive | OR‡ (95% CI) | p-value | Adjusted OR‡  (95% CI) | p-value |
| Screening Tool Score: 0 | 2 (1.2%) | 2.39  (0.56,10.18) | 0.24 | 2.23  (0.56, 8.97) | 0.26 | Screening Tool Score: 0 | 2 (0.9%) | 3.36  (0.79, 14.29) | 0.10 | 3.27  (0.78, 13.65) | 0.10 |
| 1 | 6 (1.5%) |  |  |  |  | 1 | 9 (1.8%) |  |  |  |  |
| 2 | 4 (1.3%) |  |  |  |  | 2 | 9 (3.3%) |  |  |  |  |
| 3 | 9 (7.1%) |  |  |  |  | 3 | 6 (10.0%) |  |  |  |  |
| 4 | 4 (11.8%) |  |  |  |  | 4 | 1 (50.0%) |  |  |  |  |
| 5 | 2 (28.6%) |  |  |  |  |  |  |  |  |  |  |
| 6 | 0 |  |  |  |  |  |  |  |  |  |  |
| Screening Score ≥ 1: Yes | 25 (2.9%) | 2.39  (0.56,10.18) | 0.24 | 2.23  (0.56, 8.97) | 0.26 | Screening Score ≥ 1: Yes | 25 (3.0%) | 3.36  (0.79, 14.29) | 0.10 | 3.27  (0.78, 13.65) | 0.10 |
| No | 2 (1.2%) |  |  |  |  | No | 2 (0.9%) |  |  |  |  |
| Screening Score ≥ 2: Yes | 19 (4.1%) | 3.00  (1.30, 6.91) | 0.01 | 3.37  (1.49, 7.62) | 0.004 | Screening Score ≥ 2: Yes | 16 (4.8%) | 3.17  (1.45, 6.91) | 0.004 | 3.66  (1.72, 7.79) | 0.001 |
| No | 8 (1.4%) |  |  |  |  | No | 11 (1.6%) |  |  |  |  |
| Screening Score ≥ 3: Yes | 15 (8.9%) | 7.01 (3.22, 15.27) | < 0.001 | 8.84  (4.05, 19.27) | <  0.001 | Screening Score ≥ 3: Yes | 7 (11.3%) | 6.08  (2.47, 15.01) | < 0.001 | 6.28  (2.43, 16.23) | < 0.001 |
| No | 12 (1.4%) |  |  |  |  | No | 20 (2.1%) |  |  |  |  |
| Screening Score ≥ 4: Yes | 6 (14.3%) | 7.74  (2.94, 20.35) | < 0.001 | 9.01  (3.32, 24.46) | <  0.001 | Screening Score ≥ 4: Yes | 1 (50%) | 38.85  (2.36, 639.62) | 0.01 | 67.97  (0.68, 6761.62) | 0.07 |
| No | 21 (2.1%) |  |  |  |  | No | 26 (2.5%) |  |  |  |  |
| Screening Score ≥ 5: Yes | 2 (25.0%) | 13.4  (2.57, 69.74) | 0.002 | 12.78 (2.05, 79.78) | 0.006 |  |  |  |  |  |  |
| No | 25 (2.4%) |  |  |  |  |  |  |  |  |  |  |
| *‡ Odds ratios include robust standard errors. Adjusted odds ratios include covariates for: age (continuous), sex (male/female), educational attainment (none/any primary/any secondary/beyond), married (yes/no), worked in last 7 days (yes/no), number of living children*  *^ Full tool includes: “overlooked groups,” tested ≥12 months ago or never; came in for STI; ≥4 recent health consultations; came in for malaria; ≥3 recent sexual partners; recent condomless sex with a non-stable partner. Possible score ranges 0-7.*  *^^ Reduced tool includes: “overlooked groups,” tested ≥12 months ago or never; came in for STI; ≥4 recent health consultations; came in for malaria. Possible score ranges 0-5.* | | | | | | | | | | | |
